# An Improved Method of Making the Matrix for Irregular-Shaped Porcelain Inlays

**Published:** 1902-06

**Authors:** 


An Improved Method of making the Matrix for Irregular-Shaped Porcelain Inlays.—An improved method of making the matrix for porcelain inlays is noted in C. Ash & Son's Quarterly Circular for December, 1901, page 421, the more marked novel features of which are the use of a preparation of gum lac introduced by Mr. Girdwood for taking the impression, and the use of Spence metal for the mould into which the gold or platinum matrix is swaged by means of Ash's inlay swager. The advantage claimed for this combination is greater accuracy in the impression, a more resistant mould than one made of plaster, and a saving of time, as no time is lost waiting for plaster to set. It is claimed that it only occupies eight minutes to take the impression, make the Spence metal mould, and swage the matrix.

There is no question but that a matrix swaged into a mould will produce a better-fitting inlay than one fitted to the cavity by the skilful use of burnishers, etc., provided that the mould is an exact reproduction of the cavity. A swaged matrix not only fits the mould in which it is made closer than does a burnished one the cavity in which it is formed, but the metal of the matrix is more nearly at rest, and it is therefore less liable to change form during the subsequent operations. The difficulty heretofore has been to produce a perfectly accurate mould, one that would retain its accuracy during the swaging. Plaster is far too friable to retain the extremely sharp edges, often so important in this work, while fusible metal is not only incompatible with convenient impression materials, but, used as it must be for this purpose, does not usually produce an accurate cast.

For this new process the impresion is taken upon a little impression-cup. Impression-cups made for this purpose are little metal plates of a size and shape to reach and conform to the various positions in which cavities suitable for porcelain inlays are usually found. They are perforated with a few small holes, the better to retain firmly the impression material. They usually have been made with a long handle, but this has proved a disadvantage, interfering, as it does by its leverage, with that steadiness essential to securing an accurate impression. Mr. E. B. Dowsett has devised a set of impression-cups for this purpose in which the handle is dispensed with. They are made in pairs connected together by a short pin and split tube so that when united they are firmly attached to each other and yet may be readily separated for convenience of after work. The advantage of thus connecting them is mainly in that the second cup forms a very convenient rest for the ball of the finger or thumb to press upon and hold the cup firmly and steadily in place, and thereby secure a more reliable impression.

After the impression has been taken the cup is detached from its fellow and by means of investment material is implanted, face up, in a special flask, and a cast made from it of Spence metal. Spence metal is hard and rigid, but very brittle. With proper care it reproduces very accurately the mould, more so than fusible metal, and retains its accuracy under the swaging force required remarkably well. It requires but little heat for fusion, but is very sensitive to overheating. To avoid this Mr. Carl Christensen has devised a ladle with a cup-shaped downward projection which serves to restrain the heat from reaching the sides of the ladle. When melted, the Spence metal has the appearance of a spongy mass; the heat is withdrawn when this condition is reached, and the mass stirred with a stick until it settles down and becomes clear and free from bubbles. It then stiffens somewhat and finally liquefies. This liquid condition must be looked for, and when it appears the metal must be quickly poured. The cast thus obtained is used in Ash's inlay swager precisely as would be one made of plaster.

Objection has been made to the gum lac impression compound on account of the heat required to make it sufficiently plastic.

It is contended, however, that except in very sensitive cavities this causes no more discomfort than would the use of gutta-percha as ordinarily used for fillings.

Another novelty in matrix-making is noted in the *Dental Record* (London), January, 1902, page 40, suggested by Dr. Bruhn. This is a matrix die-plate, similar to those used to form the cusps of shell crowns, with a number of depressions upon its upper surface into which the metal of the matrix is pressed and partly shaped before being adjusted to the cavity. It is said to be endorsed by Dr. Jenkins, of Dresden, as having practical value.

